# Teachers’ use of semiotic resources in the multimodal-multilingual language instruction

**DOI:** 10.1093/jdsade/enaf076

**Published:** 2026-01-16

**Authors:** Maike Beyer

**Affiliations:** Department of Linguistics, Stockholm University, Sweden; Insitutionen för lingvistik, Stockholm 10691, Sweden

## Abstract

Teaching newly immigrated deaf and hard-of-hearing multimodal-multilingual learners requires the flexible and adapted use of diverse semiotic resources. This study adopts a linguistic ethnography approach to examine how three teachers use various semiotic resources in classrooms where German and German Sign Language (DGS) are taught simultaneously. The study analyzes (a) which semiotic resources are employed and (b) how teachers jointly use them to support students’ languages learning. The findings reveal that teachers commonly draw on DGS, written (and spoken) German, and further semiotic resources: fingerspelling (manual alphabet of DGS), mouthing (German), and signing systems. Moreover, they may also incorporate student-initiated semiotic resources, i.e., students’ heritage sign language and the manual alphabet of heritage sign language, to connect these to targeted languages. Furthermore, teachers calibrate additional semiotic resources, such as digital media, into their teaching to foster understanding. Overall, the study highlights teachers’ strategies for supporting language development and facilitating understanding in a multimodal-multilingual learning environment.

## Teachers’ use of semiotic resources in multimodal-multilingual language instruction in Germany

Researchers have repeatedly highlighted the lack of adequate language education for immigrant deaf and hard-of-hearing multimodal-multilingual learners (IDML) both internationally (e.g., Cannon & Guardino, 2022; Cannon & Marx, 2023; Holmström & Sivunen, 2022) and in Germany (e.g., Begon, 2024; Begon et al., 2023; Marx & Urbann, 2022). Most of Germany’s schools for deaf and hard-of-hearing (DHH) students include IDML, who, in 2024, made up ~9.6% of the total student population at these schools (Kösler et al., 2025). However, the heterogeneity of this group, particularly regarding educational backgrounds and language acquisition histories, poses considerable challenges for adaptable language instruction.

Documented best practices for supporting IDML in multimodal-multilingual language education in Germany remain scarce. Teachers report uncertainties regarding language acquisition and development, effective teaching strategies, and choosing suitable materials for IDML (Begon, 2024), which is reinforced by the lack of curricular and instructional guidelines (Begon, 2024; Kösler et al., 2025; Marx & Urbann, 2022; see also Holmström, 2024 for Sweden). As a result, teachers commonly rely on instructional approaches and materials designed for hearing multilingual students or DHH students, which they must independently adapt or improvise to meet the specific needs of IDML (Begon, 2024; Holmström, 2024). Finally, there is almost no opportunity presented for teachers to learn necessary skills; although five German universities offer programs in deaf education, only two offer a class addressing teaching practices for IDML (Marx & Urbann, 2022). Consequently, teachers in deaf education often teach IDML without specific training to address this group’s multimodal-multilingual and didactic requirements (Begon, 2024).

Previous researchers emphasize the importance of understanding how instructional approaches and learning materials can be tailored for IDML (Marx & Urbann, 2022; Wolbers et al., 2023). However, how teachers adapt their communicative practices to students’ semiotic repertoires in multimodal-multilingual classroom settings remains unclear. For this reason, this study addresses the following research questions:

1. Which semiotic resources do teachers use while instructing IDML?
2. How are these semiotic resources jointly used to teach in multimodal-multilingual language learning classes?

For this study, data were generated from the project “Additional Language Acquisition of Deaf and Hard-of-Hearing Immigrant Students in Germany” funded by the “Innovationspool Sonderpädagogik”, which aims to explore multimodal-multilingual language education in the classroom with IDML. A linguistic ethnographic approach was chosen to identify and analyze the instructional practices in German as a second language (in German: *Deutsch als Zweitsprache—*DaZ) while teaching German and German Sign Language (DGS) simultaneously by focusing on three teachers teaching IDML at the primary and secondary school levels.

## IDML in educational settings in Germany

As outlined above, Germany represents a special situation for IDML in schools. Although most schools educate immigrant students, there is little to no support for their teachers, and few opportunities to learn how to teach them. This is especially challenging considering that IDML constitute a heterogeneous group whose language(s) experiences are shaped by both their DHH identity and their migration experiences.

First, “people who are Deaf are not Deaf in the same way” (Hochgesang, 2015, p. 22). Language acquisition and use among DHH individuals is complex due to their diverse access to sign, spoken, and written language and the different stages of life that they became DHH (Wolbers et al., 2023). For instance, in Germany, DHH individuals may use spoken German or DGS, or both, alongside written German (Becker & Jaeger, 2019). Some also use additional heritage (signed, spoken and written) languages, home signs, gestures, or spoken language supported by signs (Wolbers et al., 2023).

Second, the conditions of migration can be very different for IDML. Families might plan their move, while refugees might flee unexpectedly, leading to challenges in adapting to new educational and linguistic environments (Akamatsu & Cole, 2000; Cannon & Guardino, 2022). Globally, access to education for DHH learners is often limited (for a review of educational situations worldwide, see Moores & Miller, 2009), regardless of the school system in place. Further, an estimated 90% of DHH individuals globally have never been to school (Haualand & Allen, 2009). This lack of access can limit opportunities to acquire a named sign language, particularly in contexts where sign languages are not recognized or supported within the educational system (Holmström & Sivunen, 2022). For instance, a 9-year-old might learn DGS as their first accessible named language upon arriving in Germany. Further, communication strategies that were unproblematic in the individual’s home country may no longer suffice in a new country that privileges high proficiency in privileged named languages (Holmström & Sivunen, 2022). Previous access to languages and education then affects academic progress.

Finally, the conditions of education in the host country are different. In Germany, DHH students may attend either a special school or a mainstream school with the support of a pedagogical assistant (Günther et al., 2009). Educational placement decisions for IDML depend on both individual needs and structural possibilities and are typically made in collaboration with parents and school staff. Communication modes in educational settings tend to focus on spoken language or bimodal-bilingual education (DGS and spoken/written German), while education in DGS for DHH students remains rare (Becker, 2014). However, there is a growing tendency to promote education in DGS. For example, in the state of Nord-Rhine-Westphalia, DGS has been recognized as a mandatory language of education since August 2024 (Ministerium für Schule und Bildung NRW, 2024).

In summary, IDML enter school with highly diverse language and cultural experiences, varying previous educational experiences, and different access to education. Their communication and learning practices are shaped by these individual histories, which in turn influence their ability to learn new schooling languages and keep pace with formal education. These interconnected, complex factors make it difficult to predict the multimodal-multilingual learning needs of learners and, hence, the language development (Becker, 2014). Therefore, teachers must be attuned to their students’ “histories, orientations, intentions, thoughts, and feelings” (O’Neill, 2017, p. 107), drawing on previous experiences to adapt teaching that is multicultural—acknowledging diversity—and intercultural—promoting interaction, encounter, empathy, and shared understanding as the basis for learning (Portera, 2020). Such responsiveness to students’ backgrounds is essential for fostering understanding and supporting the development of multimodal-multilingual knowledge among IDML (Wolbers et al., 2023).

## Semiotic resources in multimodal-multilingual classroom

In discourse on communication, a holistic approach is often taken. This sees communication as inherently multimodal and multilingual, consisting of different semiotic resources used for meaning-making (Ferrara & Hodge, 2018; Henner & Robinson, 2023; Kusters et al., 2017). Since the focus is on multimodal-multilingual communication, the consideration of semiotic resources is especially useful when analyzing communication with DHH individuals. These resources can include sign languages, spoken languages, written languages, the use of objects, gestures, home signs, pointing, (digital) media, and more.

Individuals acquire semiotic resources through interpersonal communication and lived experiences, forming a unique semiotic repertoire (Blackledge & Creese, 2017; Kusters et al., 2017) as individuals learn to use resources in context-dependent ways (Ferrara & Hodge, 2018). Since students have different semiotic resources and levels of proficiency, they have individual communication needs in the classroom, which can pose challenges for teachers (Becker, 2014; for the Swedish context, Schönström & Holmström, 2021). For instance in the German context, using spoken German, spoken German with supported DGS signs, DGS signs and the manual alphabet of DGS following written German structure, or DGS as semiotic resources of instruction may not be equally accessible or comprehensible to all students in the classroom because students have varying levels of access and exposure to DGS and German (Becker, 2014; for the Swedish context, Schönström & Holmström, 2021).

During multimodal-multilingual teaching, teachers must first ensure that all students understand the instructions and, secondly, present the languages to be learned to provide a meaningful learning environment (Becker, 2014). Teachers’ ability to navigate the complex use of semiotic resources and recognize students’ background knowledge, experiences, and learning strategies in a multimodal-multilingual classroom is a crucial aspect of effective instruction (Bagga-Gupta, 2002; Cummins, 2021; Knoors & Hermans, 2012). To address the diverse communication needs in multimodal-multilingual classrooms, teachers must employ strategies to adapt to each student’s semiotic repertoire and connect with new languages to be learned. Here, concepts like calibration (Moriarty et al., 2024; Moriarty & Kusters, 2021) and translanguaging (García & Wei, 2014) have been proposed to focus on the use of different semiotic resources to adapt to different communicative needs.

Calibration is an emic concept emerging from Deaf communities and refers to languaging—the purposeful adjustment of communication, drawing on any relevant semiotic resources, whether monomodal, monolingual, multimodal, and/or multilingual, to foster mutual understanding in interaction. Translanguaging, in contrast, highlights the inherently dynamic and flexible use of resources from two or more named languages as part of meaning-making. Translanguaging can be part of calibration, but calibration’s scope extends beyond the use of multilingual resources to encompass all communicative adjustments aimed at fostering mutual understanding (Kusters, 2024; Moriarty et al., 2024).^1^ For example, in calibration practices, teachers may link the semiotic resources of the schooling language(s) (DGS, spoken German, and written German) incorporating varying degrees of mouthing from different signed and spoken languages, slower or faster fingerspelling from different sign languages, students’ heritage language(s), and adding, home gestures, gestures, drawings, or pictures to support the students’ learning process. In the case of fingerspelling, it plays a unique role in instruction by uniting two semiotic resources into one. According to Holmström and Schönström (2018), fingerspelling acts as *intramodal translanguaging*, specifically representing written German in the signed mode. Instead of switching to the spoken or written mode, written or spoken language is seamlessly integrated and visualized through the signed mode.

Building on this theoretical concept of calibration, Kusters (2024) conducted a linguistic–ethnographic study to investigate calibration strategies in classroom settings. In this study, International Sign (IS) was used as the language of instruction, in a situation with the specific content focusing on explaining the healthcare system in Denmark. IS is used when deaf individuals without a common named language come together. IS usually integrates semiotic resources such as various national sign languages and incorporates mouthing from English and other spoken languages (Kusters, 2024; for further reading on IS; see also Kusters, 2021; Kusters et al., 2024). Kusters et al. (2024) demonstrated how calibration practices draw on various semiotic resources to ensure understanding in the classroom. One illustrative example involved two teachers explaining the word *virus*, where one teacher used the manual alphabet of IS to slowly fingerspell the word V-I-R-U-S while mouthing the word in Dutch. Simultaneously, a second teacher writes the word *virus* in English on the blackboard. This coordinated use of semiotic resources underscores how teachers adapt their strategies to support understanding in the classroom (Kusters, 2024). As seen in Kusters’ study (2024), different semiotic resources related to the same concept can be calibrated simultaneously or sequentially.

The connecting of different semiotic resources of the same concept is also referred to as chaining (Tapio, 2019; Bagga-Gupta, 2002; Humphries & MacDougall, 1999). The strategy of chaining emphasizes the equivalence between different semiotic resources. For example, a teacher might point to a word on the blackboard while simultaneously signing it, hence chaining the semiotic resources from written language and sign language (Humphries & MacDougall, 1999, p. 90). Considering chaining practices as part of teaching strategies can support understanding, connecting, and comparing different semiotic resources of the same concept in multimodal-multilingual teaching.

## Method

This study examines multimodal-multilingual language teaching practices by focusing on semiotic resources in instruction IDML. It addresses the following research questions:

1. Which semiotic resources do teachers use while instructing IDML?
2. How are these semiotic resources jointly used to teach in multimodal-multilingual language learning classes?

Using a linguistic ethnography approach, this study investigates the use of semiotic resources employed by teachers in multimodal-multilingual classrooms. This approach assumes that communication is a practice shaped by cultural and social contexts (Hou & Kusters, 2019). Throughout the research process, researchers’ own backgrounds, experiences, and biases must be taken into account (Graham & Horejes, 2017). Reflexivity is central in linguistic ethnography, acknowledging that interpretations are influenced by the interactions between participants and researchers (Barwell, 2019).

In terms of positionality, I am a DHH white woman from Germany who grew up with spoken German and learned DGS as an adult. My work experience spans various educational settings, particularly language learning classes, including a DHH school, a university, and an organization for deaf and hard-of-hearing migrants. These experiences have informed my research interest in multimodal-multilingual language teaching and the use of semiotic resources in teacher–student interaction.

Three teachers from one school participated in this study: T01, T02, and T03. Table 1 provides an overview of their total teaching experience, specific experience with teaching IDML, hearing status, migration background, and semiotic resources.

**Table 1:** Overview of the observed, recorded, and interviewed teachers.

Teacher	T01	T02	T03
Field of Work	Grades 1–5, Second language class	Grades 1–5, Grades 6–11, Second language class	Grades 6–11, Second language class
Teaching experience with DHH pupils in years (in general)	22	8	5
Teaching experience with IDML in years	1	2.5	5
Hearing status	Deaf	Hard of hearing	Hearing
Migration background	Yes	No	No
Semiotic resources Sign language(s)	DGS, [heritage] sign language	DGS, IS	DGS
Written language(s)	German, [heritage] written language (1), [heritage] written language (2)	German, English	German, English
Spoken language(s)	n/a	German, English	German, English, Spanish (basic)

*Notes*. DGS (German Sign Language); IS (International Sign).

**Figure 1:**
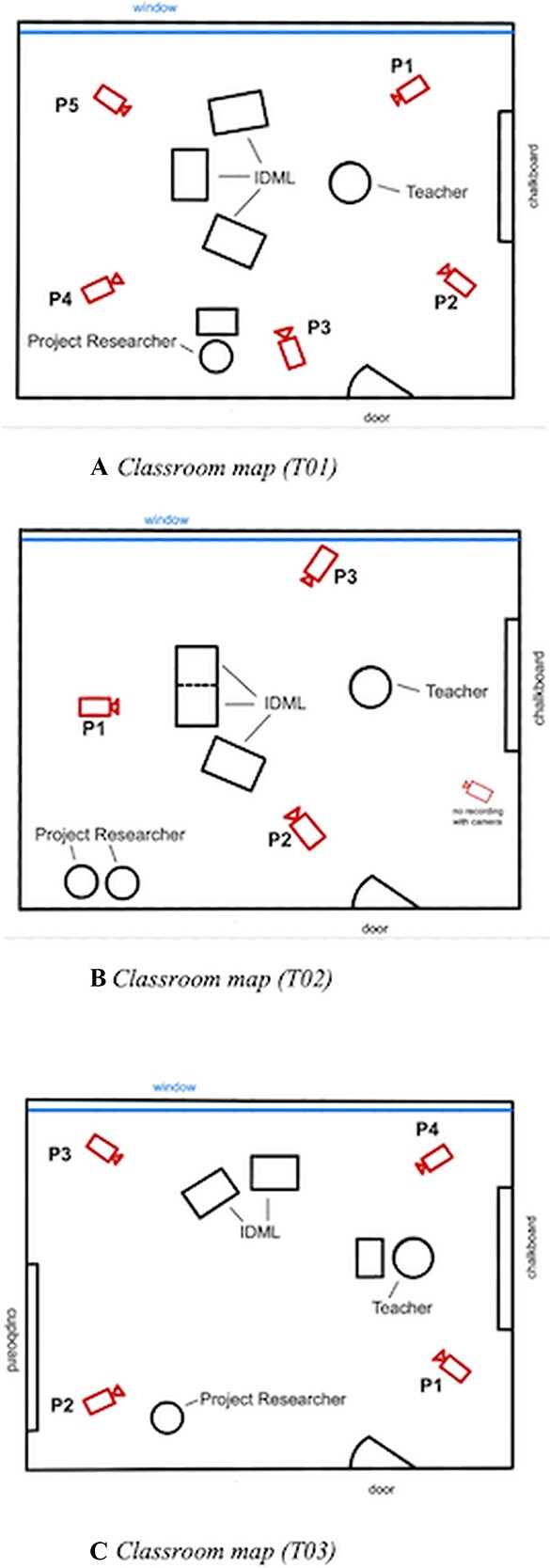
(A–C), Classroom maps.

Data were collected between March and June 2023 at a school for DHH students in Germany as part of the project “Additional Language Acquisition of Deaf and Hard-of-Hearing Immigrant Students in Germany” funded by the “Innovationspool Sonderpädagogik”. Data collection included descriptive data from field notes, classroom videography, and semi-structured interviews. The focus of data collection and analysis was on multimodal-multilingual communication in the classroom, including interactions between teachers, IDML, and their peers. No further data about the students’ background information was collected. The video-recorded data focused on DaZ instruction and captured ~4.5 classroom hours taught by the three teachers. Between three and five cameras were used to record each lesson from multiple perspectives (see Figure 1A, B, C). The project organized and conducted video recordings in the classroom. The author carried out the interviews. T01 and T02 used DGS, while T03 used spoken German as the language of the interview.

Ethical approval was granted by the Ethics Committee of the Faculty of Human Sciences at the University of Cologne. Before data collection, an information session was held for teachers, the legally authorized representative (LAR) of students, and school administrators, during which the study and consent forms were explained in detail (e.g., that participation in the study was voluntary and the rights to withdraw, restrict, or cancel at any time without disadvantage).

The introductory session of the research process was conducted in DGS, German, and, upon request, the participants’ heritage sign language, heritage spoken language, and heritage written language. The choice of languages was determined in consultation with the school administration, teachers, and the LAR. A PowerPoint presentation incorporating all important information about the research process and informed consent, with METACOM symbols (Kitzinger, 2019), was used to support the explanations of informed consent visually. These symbols are designed to be clear and easy to understand, primarily using familiar, concrete imagery from everyday life. Abstract or conceptually demanding representations are avoided; instead, concepts that cannot be depicted unambiguously are illustrated through short visual stories (Kitzinger, 2019).

Following this introduction session, consent forms were offered in following formats: in German and, upon request, in two different heritage written language of the LAR. For students, the informed consent form and presentation were provided in easy-to-read German, accompanied by METACOM symbols (Kitzinger, 2019). The consent forms were reviewed together with the students and teachers and, upon request, with the project team.

While all teaching sessions focused on language teaching, the specific content varied. T01 and T02 taught the same three IDML, while T03 taught two different IDML. Students who attended the lesson in this study varied in age, school levels, semiotic repertoires, and language(s) preferences. Table 2 provides an overview of the data used in this study.

**Table 2:** Overview of the data used in this study.

Teacher	T01	T02	T03
Interview	00:42:07 h	00:33:08 h	00:40:05 h
Classroom video-recordings	00:37:48 h	01:19:22 h	02:33:46 h
Data analysis	Phenomena and patterns of categories and refined categories through ELAN (ELAN, 2022) based on the following data: Field notes Interview Video-recordings		
Students (IDML)	3	3	2
Perspectives (P)	5	3	4
Goal	To analyze which semiotic resources teachers use and how teachers connect these semiotic resources to create understanding during language(s) teaching practices with IDML		

*Notes*. IDML (Immigrant deaf and hard-of-hearing multilingual learners).

The data were annotated using ELAN (EUDICO Linguistic Annotator), a multimedia annotation software (ELAN, 2022). All annotations were carried out by the author of the article. The analysis was conducted through repeated checks with the recordings at different stages by the author, ensuring the reliability of the annotated semiotic resources.

The annotation followed the four analytical categories developed by Holmström and Schönström (2018)—language, mode, interaction, and pointing. Since languages can be expressed through different modes, language and modes were kept on different tiers. Interaction was included to capture teachers’ engagement with media and materials. Pointing covered nonlinguistic components of sign languages. As pointing is recognized as a pedagogical tool, pointing was annotated if different semiotic resources were chained to create joint attention (Holmström & Schönström, 2018). These analytical categories served as initial criteria for identifying semiotic resources in teacher–student interactions in the video recordings. After which, the categories were classified and refined inductively based on teachers’ use of semiotic resources emerging from the data. The refined categories in ELAN (2022) are presented in Table 3.

**Table 3:** Categories and annotation form refined in ELAN (2022).

Categories		Annotation
DGS Students’ Heritage Sign Language Sign language (both)	Lexical sign Lexical sign	SIGN [SIGN]
	Indicating sign	Active hand (a.h.): pointing Passive hand (p.h.): handshape
Fingerspelling (both)		F-I-N-G-E-R-S-P-E-L-L-I-N-G
Mouthing	Mouthing is borrowed from spoken language	M-typ
	Adverbial expression, constructed action congruent, editorial	W-typ
Spoken German		Spoken
Written German		*Written*
International Sign		[IS-SIGN]
(Digital) Media		(Digital) Media x
Object		Object x

*Notes*. DGS (German Sign Language). IS (International Sign). a.h. (active hand). p.h. (passive hand).

The table is there to demonstrate that written language is in italics.

The annotated semiotic resources used by teachers were analyzed to identify recurring phenomena and patterns. The focus was on sequences where teachers used more than one semiotic resource to identify phenomena and patterns during instruction. Furthermore, background interviews and field notes were involved and analyzed to contextualize teachers’ use of semiotic resources.

In the results section, lexical DGS signs are represented in English capital letters (i.e., SIGN), and indicating signs are shown, i.e., active hand (a.h.): pointing; passive hand (p.h.): handform. German spoken words are shown in standard English written notation (i.e., spoken word), whereas written words are shown cursive (i.e., *written word*). Students’ heritage language will also be presented in square brackets along with its English translation (i.e., [SIGN]). Dashes in the gloss indicate that fingerspelling is used (i.e., F-I-N-G-E-R-S-P-E-L-L-I-N-G). If the focus is on mouthing, notation is represented with *M-type*, meaning that mouthing is borrowed from spoken German. If the mouthing is represented with a whole face gesture (i.e., adverbial expression, constructed action congruent, editorial), this is noted as *W-type* (see categorization of mouth actions developed by Crasborn et al., 2008; adapted by Mesch & Schönström, 2021).

The description of classroom instruction focuses on the choice of semiotic resources the teachers use. Quotes from the interview are based on translations by the author and are aimed at emphasizing what was said. Below, this study focuses on the teachers’ chosen semiotic resources during instruction, focusing not on differences among teachers but on common recurring phenomena and patterns used by teachers.

## Results

As mentioned above, this study identifies and analyzes three teachers’ semiotic resources and how these teachers jointly used different semiotic resources in teaching IDML. The results present illustrative examples with a focus on teachers’ use of semiotic resources. The students’ semiotic repertoires and communication preferences play a role in shaping these practices.

The results are structured into two categories and further subcategories: (1) commonly used semiotic resources, including DGS, written (and spoken) German. This category is divided into further sub-categories: (a) fingerspelling (DGS manual alphabet), (b) mouthing (German), and (c) DGS signs, fingerspelling, and written German (signing system), (d) spoken German and DGS signs (signing system). The second category includes (2) calibration with the sub-categories (a) student-initiated semiotic resources and (b) further teacher-initiated resources.

These categories and identified semiotic resources used by teachers during languages instruction with applied context are demonstrated in Table 4. These categories are demonstrated and explained in this section, accompanied by illustrative examples of classroom interactions and excerpts from teacher interviews, to provide further insights into the semiotic resources used.

**Table 4:** Results of categories of identified semiotic resources and strategies during teachers’ language instruction.

Categories	Semiotic resources	Applied context
(1) Commonly used semiotic resources	DGS Written German Spoken German	Teacher-initiated semiotic resources to teach targeted languages
	a) Fingerspelling (DGS)	Visualize the written word in the signed mode and chain it to the written word and DGS sign
	b) Mouthing (German)	Component to express the full linguistic features of a lexical DGS sign Support memorization of the written word
	c) DGS signs, fingerspelling (DGS) following written German (signing system)	Visualize written German in the signed mode
	d) Spoken German with DGS signs (signing system)	Support keywords with DGS signs
(2) Calibration		
(i) Students’ initiated semiotic-resources used by teacher	Heritage sign language Heritage written language IS ASL English^2^ Fingerspelling (heritage sign language) Mouthing (heritage language)	Students-initiated semiotic resources taken up by teachers to connect with targeted languages to foster understanding in interaction
(ii) Further teacher-initiated semiotic resources	Pictures Gestures^3^ Simple signs Role-plays^3^ Movements^3^ Objects etc.	Teacher-initiated semiotic resources to ensure that all students understand the instruction

*Notes*. DGS (German Sign Language). IS (International Sign). ASL (American Sign Language).

### 1) Commonly used semiotic resources: DGS, written (and spoken) German

The first category addresses the “commonly used semiotic resources” employed by the teachers. According to the video recordings, teachers T01 and T02 used semiotic resources DGS and written German, while T03 also incorporated spoken German. During interviews, teacher T01 emphasized the two languages’ learning objectives in teaching the subject DaZ.

My teaching goal is clear, German Sign Language. This is the goal. I teach the students who come to Germany in DGS. It is about teaching students new to Germany to become familiar with the German language, for example, through aspects of German culture and understanding, which language is currently being used. The students aim to possibly attend, for example, in training or at the final exam before graduation, at study at university, and at work in the future. Hence, the aim is to teach and learn German Sign Language and additional DaZ, German as a Second Language. This is also a goal during teaching. DaZ is important, for example, for writing, for letters, for advertisements, for reading, or subtitles on the TV, both are important goals. For this reason, I teach both German Sign Language and German. Both languages are the focus of my teaching.

T01 and T02 shared the same instructional goal for the three IDML (Students 1, Student 2, and Student 3) to facilitate the acquisition of DGS and written German. As observed in classroom interaction, the three students have similar semiotic resources of their heritage sign language in their semiotic repertoires. In contrast, T03’s goals vary by student: Student 4 learns spoken/written German and DGS signs, while Student 5 focuses on DGS and written/spoken German. As T03 noted in the interview, communication between the students can be challenging, as each student has different semiotic repertoires and distinct language learning goals.

As observed in classroom recordings and according to the interviews, during DaZ instruction, IDML are taught two new target languages, DGS and German, which include semiotic resources: DGS, written German, and spoken German. In addition to different languages and modes, i.e., DGS and (spoken and written) German, as languages of instruction, teaching strategies also included further semiotic resources: (a) fingerspelling (DGS manual alphabet), (b) mouthing (German), (c) DGS signs, fingerspelling of the DGS manual alphabet and written German (signing system), and (d) spoken German and DGS signs (signing system) to support students’ language development in German in the signed mode and aspects of DGS.

#### a) Fingerspelling (DGS manual alphabet)

Fingerspelling of the DGS manual alphabet was a common semiotic resource used by all three teachers. The written word was commonly introduced and explained with DGS, and then the word was fingerspelled by staying in the signed mode to demonstrate written German. Noticeably, although fingerspelling is used in the signing mode, fingerspelling is uncommon in DGS (Hillenmeyer & Kleyboldt, 2020).

An illustrative example showed how T02 explained two similar-looking words that differ in meaning—*schlafen* “sleep” and *verschlafen* “oversleep”—using three semiotic resources: written German, DGS, and the manual alphabet of DGS.

The teacher wrote *schlafen* “sleep” on a tablet connected to the classroom screen. Student 2 mouthed (M-typ) schlafen “sleep” but signed STREET in DGS. The teacher shook her head, asked if the students knew the word, and clarified by fingerspelling SCH-L-A-F-E-N “sleep”. Student 1 responded with the DGS sign PAST to indicate that the word had been covered before, then signed KNOW in DGS and the International Sign YES, and produced an unclear sign. The teacher pointed to the screen and prompted again. Again, Student 1 produced an unclear sign, which the teacher interpreted as WALK. Student 2 repeated WALK but accompanied it with the mouthing (M-typ) of schlafen “sleep”. The teacher then provided the DGS sign SLEEP. Student 2 acknowledged this by signing that they always forget this sign.

To extend the explanation, the teacher wrote *schlafen* “sleep” and a blank prefix line before and asked what was missing in the blank line. Student 3 began to fingerspell the word *schlafen* “sleep” and the teacher affirmed but pointed to the missing prefix. The teacher then signed OVERSLEEP in DGS and wrote *ver-* on the board. Student 2 recognized the explanation, mouthed (M-typ) *Achso* “Oh, I see”, and nodded in agreement. T02 then described the meaning of OVERSLEEP in DGS (see Excerpt 1). After Student 3 attempted to fingerspell V-E-R-SCH-A-L-F-E-N “oversleep”, which the teacher corrected to V-E-R-SCH-L-A-F-E-N “oversleep”. Finally, the teacher contrasted SLEEP and OVERSLEEP by repeating their fingerspelling and illustrating the difference with an everyday example, ensuring that students understand the lexical contrast.

**Excerpt 1:** Explanation of German written word in DGS.

	T02	
1	written German	*oversleep*
2	DGS	OVERSLEEP (M-type) MEANING DIFFERENCE WHAT? SLEEP (W-type) YOU KNOW NORMAL SLEEP (M-type) BUT I FINISH OVERSLEEP (M-type) MEANING LATE COME PURPOSE I FINISH OVERSLEEP (W-type) ALARM BLINK+++ I ALARM TURN-OFF I FINISH SLEEP (W-type) AGAIN I LATE COME “What is the difference in meaning between oversleep and sleep? You know what sleep normal is, but oversleep means I was late because I overslept. My alarm light flashed, I turned it off, went back to sleep, and then I arrived late.”
3	Fingerspelling (DGS)	V-E-R-SCH-L-A-F-E-N ‘oversleep’
3	Mouthing (German)	M-typ (oversleep)

*Notes*. The mouthing description focuses explicitly on SLEEP and OVERSLEEP.

DGS (German Sign Language).

In conclusion, in this interaction, Student 1 signaled knowledge of the word but struggled to produce the corresponding sign. Student 2 recognized the mouthing of the word while producing mismatched signs, made further guesses, and then seemed to understand the teacher’s explanation of the distinction between schlafen “sleep” and verschlafen “oversleep”. Student 3 contributed by fingerspelling both words, although with errors, and actively participated in the correction process guided by the teacher. This example shows how T02 explained new words to the students using three newly learned semiotic resources: written German, DGS, and fingerspelling.

#### b) Mouthing (German)

Furthermore, as seen in Excerpt 1, explicit mouthing was employed in the instructions. In the interview, T01 shared insights into using mouthing and emphasized the importance of its use.

The role of mouthing is central. It is not about improving spoken articulation (...) but about mouthing to support signing. Without clear mouthing, the signs become less understandable. Why is this extra important? When mouthing is known, it helps memorize the corresponding written words, which supports vocabulary learning in written language. (...) Mouthing helps in reading and understanding words and is also valuable in (finger)spelling—an advantage for learning.

Explicit mouthing (M-type) was observed during a learning exercise where the teacher taught food-related vocabulary. The exercise incorporated cards with written words and pictures illustrating their meanings. Mouthing as a semiotic resource was observed with two different functions.

First, the teacher used mouthing to respond to students’ lexical signs, who do not use mouthing (M-typ). The teacher repeated the sign along with mouthing to encompass the entire linguistic expression of the sign. This illustrative example was observed in classroom recordings by T02. Student 3 signed APPLE without mouthing, and the teacher responded by repeating the sign APPLE with the corresponding mouthing (M-type) of Apfel “apple”. Second, teachers T01 and T02 used explicit mouthing in various situations while students spelled words using fingerspelling. For example, in an exercise led by T01, one student was required to express a DGS sign, while their peers had to express the same word using fingerspelling of DGS. While students fingerspelled to the corresponding sign, the teacher concurrently mouthed (M-type) to the fingerspelled letters. For example, the student fingerspelled the word T-O-M-A-T-E “tomato” while the teacher simultaneously mouthed (M-type) it. This analysis underlines mouthing as a semiotic resource in teaching instruction used by teachers, encompassing spoken/written German.

#### c) DGS signs and fingerspelling and written/spoken German (signing system)

The following observed patterns focus on the combined use of semiotic resources (signing systems) used by teachers, which illustrate how the written or spoken German structure is combined with DGS signs and fingerspelling during instruction. In these data, I identified the use of DGS signs and fingerspelling following the German grammar structure, also referred to as signed exact German (Becker & Jaeger, 2019; Holmström & Schönström, 2018). Signed exact German uses signs for every spoken or written word and incorporates fingerspelling to represent grammatical elements, visually aligning spoken/written German with signs of the DGS lexicon, which means three semiotic resources (written German, DGS signs, fingerspelling) are used during signed exact German. In Excerpt 2, T01 employs DGS and fingerspelling to teach a student the grammatical features of written German. The task on the worksheet is designed to practice forming sentences with food vocabulary. To demonstrate written German, the teacher used signed exact German to explain how to form the plural, using the example of AUTO “car”. This example illustrates how the written modality can be devised to make written language visually accessible through the use of the signed mode.

**Excerpt 2:** DGS signs and fingerspelling (DGS) following written German structure (signed exact German).

	T01	
1	DGS sign	EXAMPLE
2	Fingerspelling (DGS)	D-A-S “the”
3	DGS sign	EXAMPLE CAR
4	Fingerspelling (DGS)	A-U-T-O “car”
5	DGS sign	MANY
6	Fingerspelling (DGS)	A-U-T-O-S “cars”

*Notes*. DGS (German Sign Language).

#### d) Spoken German and DGS signs (signing system)

In contrast, spoken German and DGS signs, also known as signed supported German, emphasize keywords through lexical signs (Becker & Jaeger, 2019). In Excerpt 3, T03 used spoken German with DGS signs to varying ratios to explain the next work assignment to student 5. Additionally, the teacher also had the worksheet in her hand, which she referred to during the explanation. T03 taught Student 5 the vocabulary of colors and clothing items. On the worksheet, a black-and-white silhouette of a person was visible. The task involved coloring the silhouette of the woman according to the template. The teacher held the covered template and explained to the student which colors corresponded to the woman’s clothing. While the teacher explained, Student 5 pointed toward the worksheet.

**Excerpt 3:** Spoken German with DGS signs (signed supported German).

	T03				
1	spoken German	Yes, this is the women. And now we have to think about how she is			
1	DGS sign	WOMEN	THINK		
2	spoken German	dressed, the women. Here she is black and white, boring. She would			
2	DGS sign	POINTING	BLACK	WHITE BORING	
2	Media	worksheet			
3	spoken German	like to have some color. Now we have to think about how to			
3	DGS sign	LIKE	COLOR	THINK	HOW
4	spoken German	paint her.			
4	DGS sign	PAINT			

*Notes*. DGS (German Sign Language).

Through this explanation, keywords are emphasized through DGS signs. In this case, not all linguistic information (grammatical structures and words) in spoken German was visually accessible through DGS signs.

T01 and T02 thus used signed exact German to make grammatical structures of written German visually accessible. By comparison, with signed supported German, some linguistic information—specifically, grammatical information from spoken/written German—may be lost if the visual channel is used exclusively and auditory perception is limited (see Excerpt 3).

### 2) Calibration

The second category of the results is *calibration*, which shows how teachers jointly use semiotic resources to create understanding and connect these to targeted languages. This category is divided into (a) *student-initiated semiotic resources* and (b) *further teacher-initiated semiotic resources*.

#### i) Student-initiated semiotic resources

In addition to teachers’ deployment of commonly used semiotic resources, the data also reveal how students draw on their semiotic repertoires, which are recognized and incorporated by teachers. A prevalent pattern in teacher-student interaction that was identified was how teachers integrate students-initiated semiotic resources with the schooling languages, DGS and written German. T01 and T02 observed students using their heritage sign language, IS or American Sign Language (ASL). T02 also reported their approach to leveraging students’ semiotic resources, adapting to their language choices, and facilitating their transition to DGS.

I sometimes notice that students use their heritage language during exchanges. I then incorporate that language and explain how it is signed in DGS. Occasionally, I also observe students using IS or ASL during exchanges. However, it is common for young people to use various sign languages; for instance, they encounter them frequently on social media platforms like Instagram, Twitter, TikTok, or Facebook.

T03 furthermore stated that one student can effectively translate the heritage language through a translator into written German. Additionally, spoken English can be used as an additional semiotic resource during instruction.

The student falls back on their heritage written language and then partly translates texts or translates individual words because the student can simply translate that well and is also relatively fit in dealing with the iPad. And English is also a form of communication that you can choose when it is difficult in German.^2^

Excerpt 4 demonstrated an observed classroom situation. In this situation, the teacher used three semiotic resources: heritage sign language, DGS, and fingerspelling combined with mouthing. In the classroom, students sometimes employed their heritage sign language during interactions with peers and instructors. For instance, an illustrative example was the student-initiated semiotic resources of the heritage sign language that the students 1, 2, 3, and T01 had in common. T01 observed Student 1 signing the term [EASY] in the shared heritage sign language. After the subtask was finished and discussed, T01 asked the students how to sign [EASY] in DGS. Students 2 and 3 immediately produced and repeated the DGS sign SIMPLE. Consequently, the teacher facilitated the translation of this term into DGS, offering two sign variants, SIMPLE with mouthing (W-type) and EASY with mouthing (M-type). Student 1 looked at classmates, then to the teacher, and co-produced with T01 the sign SIMPLE. Subsequently, the teacher added the fingerspelling L-E-I-C-H-T “easy” with mouthing (M-type) followed by the lexical sign EASY with mouthing (M-type). Then Student 1 signed UNDERSTAND and subsequently produced the sign [EASY] of the students’ heritage sign language. This sequence illustrates how T01 connects students’ heritage sign language with DGS and written German through fingerspelling of DGS and mouthing (M-type; W-type).

**Excerpt 4:** Incorporating students’ semiotic resources.

	T01	
1	Heritage sign language	[EASY]
2	DGS	UNDERSTAND I GERMAN SIGN HOW? “I understand this sign but how is it signed in German?”
3	DGS	SIMPLE OR EASY
3	Mouthing	W-type M-type (easy)
4	Fingerspelling (DGS)	L-E-I-C-H-T “easy”
4	Mouthing (German)	M-type (easy)
5	DGS	EASY
5	Mouthing (German)	M-type (easy)

*Notes*. DGS (German Sign Language).

Another illustrative example was found in the following sequence, where one student was asked to produce the DGS sign of a word written on a card with a corresponding pictogram, while others had to identify the written word by using the manual alphabet of DGS. In this sequence, Student 3 first produced the DGS sign CHOCOLATE. Student 2 repeated this sign and then provided the equivalent in their heritage sign language [CHOCOLATE]. In the meantime, Student 1 repeated the sign CHOCOLATE hesitantly with one hand but remarked that the student had forgotten it. Then, Student 2 repeated the heritage sign [CHOCOLATE]. T01 responded and asked how to sign [CHOCOLATE] in DGS. Student 2 produced CHOCOLATE and received confirmation from the teacher. Student 2 then began to fingerspell, but both Student 1 and Student 2 struggled to fingerspell the German written word. Then the students looked at the card with the written word and the corresponding pictogram. Student 2 provided the manual alphabet of the heritage sign language and spelled [C-H-O-C-O-L-A-T-E].

Meanwhile, to support the students, T01 pointed to the word card and explained in DGS that the written word *Schokolade* “chocolate” is fingerspelled, mouthed, and pronounced in almost the same way in the students’ heritage language. Student 1 missed this part while looking at the card. Then, Student 2 successfully fingerspelled SCH-O-K-O-L-A-D-E “chocolate” twice. Student 3 then attempted to fingerspell the whole word but misspelled it. To support Student 3, Student 2 used the manual alphabet of the heritage sign language [C-H-O-C-O-L-A-T-E]. Student 3 responded with [YES] and completed the fingerspelling of the written German word using the DGS manual alphabet.

In conclusion, this phenomenon illustrates how T01 and the students used several semiotic resources: written German word, DGS sign, sign of heritage sign language, the heritage written language through the manual alphabet of heritage sign language, manual alphabet of DGS, and pictogram. This illustrates how the heritage language and the manual alphabet of the heritage sign language may serve as semiotic resources for supporting students’ development of written German.

#### ii) Further teacher-initiated semiotic resources

In addition to utilizing student-initiated semiotic resources, the teachers also draw on further semiotic resources. During the interview, the teachers reported strategies for clarifying content in the multimodal-multilingual classroom context, thereby enhancing understanding within the classroom. For instance, T02 explained:

Then I check which students have understood my input and which have not. If students have not yet understood everything, I explain it again and then, for example, add more pictures and use gestures, simple signs, or role plays to clarify the content. I decide beforehand whether I give a purely verbal lecture at the beginning or whether I additionally use movements, incorporate role-plays, or work with pictures to supplement my explanations. This is additional information that the students receive in addition to the linguistic input. That’s what I mean by double explanation.^3^

While the practice of calibration, as coined by T02 as “double explanation”, specifically highlights the conscious decision to provide an alternative or reinforced explanation when the initial input is insufficient for some students. In this sense, the practice involves recalibrating the explanation by incorporating additional semiotic resources, such as pictures, gestures, simple signs, role-plays, and movements, to ensure that all students can access the content.

An illustrative example was found in the following sequence: during instruction, T02 explained similar-looking written words. Excerpt 5 focuses on how T02 explained the written word *Knochen* “bone”. Firstly, T02 introduced the word by writing the word *Knochen* “bone” on a tablet connected to the classroom screen. Student 2 hesitated, signed in DGS FORGET, and paused in thought, while Student 1 reacted with a skeptical facial expression. T02 then signed BONE and repeated the same sign both with mouthing (M-type). In parallel, Student 2 signed with the active hand on the passive hand to indicate the hand bone (a.h.: pointing, p.h.: five-hand). T02 subsequently mirrored the student-initiated semiotic resource. Student 2 then produced the sign in their heritage sign language [BONE] toward Student 1, who replied with the DGS sign KNOW. Finally, T02 used a search engine to display images of bones, visually reinforcing the concept.

**Excerpt 5:** “*Double Explanation*” —Use of search engine of images during language teaching practice.

	T02	
1	written German	bone
2	DGS	BONE
2	Mouthing (German)	M-type (bone)
3	DGS	BONE
3	Mouthing (German)	M-type (bone)
4	DGS (indicating sign)	a.h.: pointing p.h.: five-hand
4	Mouthing	n/a
5	Digital Media	Use of images alongside the German word of bone

*Notes*. DGS (German Sign Language).

Excerpt 5 illustrates how T02 calibrates several semiotic resources: written German, DGS, then mirrors student-initiated semiotic resources and initiates the digital media of a search engine, to convey meaning for students during vocabulary instruction.

## Discussion

Building on instructional practices, the study reveals insights into semiotic resources that teachers use to support IDML’s language development. While no instructional guidelines for choosing specific semiotic resources were reported, the data show that the semiotic resources that teachers used are based on experience-driven and intuitive strategies from multimodal-multilingual classroom practices.

In DaZ classes, IDML must learn two new languages simultaneously, DGS and (written and spoken) German, alongside additional semiotic resources. These include fingerspelling, mouthing, and signing systems. Furthermore, the teachers employed a range of diverse semiotic resources, i.e., student-initiated semiotic resources (i.e., heritage sign language, fingerspelling, and mouthing of heritage language; IS; English) and digital media (i.e., pictures).

As illustrated in Excerpts 1 and 4, fingerspelling is commonly used to teach written German vocabulary through the signed modality. As well, awareness of fingerspelling is also related to reading competence and can support students’ literacy development (Lederberg et al., 2019). However, IDML must learn both the manual alphabet of DGS and the corresponding German written forms. This can make learning fingerspelling particularly challenging, especially when newly introduced words are fingerspelled rapidly (Kusters, 2024). As shown in Excerpt 1, the teacher addressed this challenge by writing out the word, explaining it, and finally fingerspelling it. Kusters et al. (2024) similarly observed that teachers chose to write out words because they assumed this would increase accessibility for students. IDML may benefit from structured support when learning how to use fingerspelling as a semiotic resource. Lee and Secora (2022), for example, demonstrate various ways in which fingerspelling can be used in language learning settings to support students. Furthermore, fingerspelling can enable IDML to communicate with individuals who use fingerspelling as a shared semiotic resource for creating meaning (Lee & Secora, 2022).

Mouthing (M-type) as a semiotic resource in language teaching is also highlighted in the findings. It is an integral feature of DGS, developed through contact between spoken and signed language (Papaspyrou et al., 2008). According to Ebbinghaus and Heßmann (1995), 86.2% of sentences with more than one sign include at least one mouthed word, and it appears to be functionally significant in DGS (Ebbinghaus & Heßmann, 1995). Hence, when mouthing is absent, signing may appear unnatural or lack fluency (Fontana & Ferrara, 2019, for Italian Sign Language). For this reason, mouthing seems important in supporting language development in DaZ instruction. However, there is little research regarding the mouthing development in DGS of DHH students in general, and especially none of IDML. Further research is needed to explore the development of mouthing as a semiotic resource in a language-learning context, explicitly examining how it is taught and learned by IDML.

Teachers also used signing systems (signed exact German and signed supported German) during instruction. Regarding whether signed supported German is used, it is important that all students have equal access to all semiotic resources; spoken language must especially be reflected (De Meulder et al., 2019). During teaching, it is important to acknowledge that “signing systems are less comprehensible to learners who rely upon signs rather than speech” (Scott & Henner, 2021, p. 123). Signing systems can be supportive, but they may also cause confusion due to their lack of consistency, standardization, and unintentional involvement of sign language grammatical features (Scott & Henner, 2021). Moreover, signing systems are commonly used in schools without empirical evidence to enhance learning (Müller De Quadros, 2018; Scott & Henner, 2021). In the present study, teachers used signed exact German to visualize written German in the signed mode with DGS signs and fingerspelling, and thus to reflect all linguistic information shared visually. In contrast, signed supported German runs the risk of omitting key linguistic information for written German. However, while semiotic resources such as fingerspelling or mouthing were intentionally used to support language development of spoken/written German, the use of signing systems appeared less systematically grounded. In some cases, their use reflected a pragmatic trial-and-error approach in the absence of instructional guidelines and evidence-based practices.

Teachers in this study generally used semiotic resources dynamically and flexibly, often without explicitly naming or describing them during instruction. Nevertheless, different semiotic resources were often combined, for instance, when using signed supported German or signed exact German. To follow teachers’ instructions, IDML need competence in DGS, spoken or written German, or fingerspelling, as well as the metalinguistic awareness to differentiate between these resources. However, this can be challenging for students who are still developing their semiotic resources in DGS, written and/or spoken German, and their ability to distinguish between different resources (Becker, 2014). IDML must become aware of the various semiotic resources, including DGS, spoken and/or written language, mouthing, and fingerspelling, and gain the competence to differentiate between signing systems and DGS. This ability supports the effective use of semiotic resources to create shared understanding among individuals with different semiotic repertoires. However, to cultivate metalinguistic awareness, students need additional structured, explicit instructions to help them distinguish among semiotic resources effectively (Plaza-Pust, 2004). Given the heterogeneity of students’ semiotic repertoires, integrating their diverse semiotic backgrounds, spanning both spoken and signed languages alongside visible semiotic resources, can enhance learning (Wolbers et al., 2023). Consequently, teaching DGS and German simultaneously requires a didactic approach that addresses the continuum of sequential and simultaneous structures (see Wolbers et al., 2023, for an example using ASL and English). This underscores the role of curricular frameworks and adapted instructional methods in classrooms with DHH students (Plaza-Pust, 2004).

Furthermore, findings reveal how teachers pick up students’ semiotic resources and connect them with targeted semiotic resources. As Holmström and Schönström (2018) stated, instruction content can only be understood if the semiotic resources are shared. For instance, T01 has a migration background and shares both the heritage sign language and the heritage written language with the students. For example, as seen in the sub-category *student-initiated semiotic resources*, T01 can draw on students’ semiotic resources in their heritage sign language and link these resources to the target languages being taught. This background enables the teacher to strengthen the connection between students’ semiotic repertoires (i.e., heritage sign language) and the schooling languages. Teachers’ familiarity with students’ heritage sign and spoken/written languages is a key asset in IDML classroom contexts. Drawing on students’ previously known semiotic resources and connecting them with the targeted semiotic resources can lead to more effective instruction in multimodal-multilingual classrooms (Cummins, 2021). In addition, strategies such as incorporating students’ semiotic resources, using (digital) media, or applying “double explanations” can further foster understanding and support knowledge building in these settings. Also, Cannon & Guardino (2022), Schönström and Holmström (2021), and Wolbers et al. (2023) stated that particular visually oriented semiotic resources, such as role-play, the use of drawings, and showing pictures/videos, should be implemented to communicate and create meaning with DHH students, including IDML. Presenting information using multiple semiotic resources enhances the likelihood of all recipients comprehending the information in DGS and spoken/written German. This leads to improved understanding and learning across different semiotic resources.

In conclusion, considering accessible communication for everyone in the classroom and developing metalinguistic awareness in multimodal-multilingual classrooms pose challenges. Teachers must effectively calibrate the diverse needs of students, which arise from their varying language acquisition and development conditions, while simultaneously instructing two new languages. Dostal and Wolbers (2014) emphasized the necessity for teachers to exhibit both skill and flexibility in communication and teaching in a multimodal-multilingual classroom. To be skilled in facilitating understanding and chaining it to rich academic content constitutes an essential competency to enhance students’ learning capabilities. Observing and documenting the use of semiotic resources in depth can be valuable for recognizing strategies and used semiotic resources during language teaching. Recognizing and responding to the diverse semiotic repertoires of IDML is not only a pedagogical challenge but a key to equitable and accessible education for this under-researched learner population. Future research should thus evaluate the impacts of the use of various semiotic resources on language acquisition, development, or promising support measures.

## Endnotes

1. Historically, this approach also resonates with the philosophy of Total Approach (also referred to as Total Communication), introduced by Holcomb at the 13th International Congress on the Education of the Deaf, which advocates for the use of all available communicative resources to ensure that DHH learners can meaningfully engage with the input they receive (Holcomb, 1972; Mayer, 2016). However, over time, the term Total Approach became a concept of confusion, as the original student-centered philosophy was increasingly reduced to a simultaneous use of speech and signing, often equated with Signing Exact English (Holcomb, 2016).
2. Note that using heritage written language and spoken English, as mentioned in the semi-structured interview, was not captured in the video recordings.
3. Note that not all *“double explanations”* mentioned in the semi-structured interview are captured in the video recordings (e.g., role plays).
